# Effect of Metabolic Syndrome on Mitsugumin 53 Expression and Function

**DOI:** 10.1371/journal.pone.0124128

**Published:** 2015-05-07

**Authors:** Hanley Ma, Jason Liu, Zehua Bian, Yuqi Cui, Xinyu Zhou, Xuefeng Zhou, Bo Zhang, T. M. Ayodele Adesanya, Frank Yi, Ki Ho Park, Tao Tan, Zhishui Chen, Hua Zhu

**Affiliations:** 1 Department of Surgery, Davis Heart and Lung Research Institute, The Ohio State University, Columbus, Ohio, United States of America; 2 Olentangy Liberty High School, Powell, Ohio, United States of America; 3 Dublin Jerome High School, Dublin, Ohio, United States of America; 4 Institute of Organ Transplantation, Tongji Hospital, Tongji Medical College, Huazhong University of Science and Technology, Wuhan, Hubei, P.R. China; East Tennessee State University, UNITED STATES

## Abstract

Metabolic syndrome is a cluster of risk factors, such as obesity, insulin resistance, and hyperlipidemia that increases the individual’s likelihood of developing cardiovascular diseases. Patients inflicted with metabolic disorders also suffer from tissue repair defect. Mitsugumin 53 (MG53) is a protein essential to cellular membrane repair. It facilitates the nucleation of intracellular vesicles to sites of membrane disruption to create repair patches, contributing to the regenerative capacity of skeletal and cardiac muscle tissues upon injury. Since individuals suffering from metabolic syndrome possess tissue regeneration deficiency and MG53 plays a crucial role in restoring membrane integrity, we studied MG53 activity in mice models exhibiting metabolic disorders induced by a 6 month high-fat diet (HFD) feeding. Western blotting showed that MG53 expression is not altered within the skeletal and cardiac muscles of mice with metabolic syndrome. Rather, we found that MG53 levels in blood circulation were actually reduced. This data directly contradicts findings presented by Song et. al that indict MG53 as a causative factor for metabolic syndrome (*Nature* 494, 375-379). The diminished MG53 serum level observed may contribute to the inadequate tissue repair aptitude exhibited by diabetic patients. Furthermore, immunohistochemical analyses reveal that skeletal muscle fibers of mice with metabolic disorders experience localization of subcellular MG53 around mitochondria. This clustering may represent an adaptive response to oxidative stress resulting from HFD feeding and may implicate MG53 as a guardian to protect damaged mitochondria. Therapeutic approaches that elevate MG53 expression in serum circulation may be a novel method to treat the degenerative tissue repair function of diabetic patients.

## Introduction

Metabolic syndrome encompasses a series of risk factors that imperil the individual in developing cardiovascular diseases and an assortment of other health problems, including type II diabetes and stroke[1–4]. A serious medical ailment that plagues a significant portion of the human population, it is not an adversity to be taken lightly. Although metabolic disorders primarily induce defects in nutrient regulation, their burden on the human body extends to various other physiological systems[1,2,4]. A less explored complication of metabolic syndrome is its impact on tissue repair; it has been widely recognized that injuries of diabetic patients are slow to recover[5,6]. One of the most notable instances of defective tissue healing in patients exhibiting metabolic disorders includes diabetic foot ulcers[5,6].

The molecular mechanisms underlying tissue regeneration were largely undefined until the recent discovery of mitsugumin 53 (MG53) by Cai et al. in 2009[7]. Chiefly expressed in hyperactive cells exposed to constant physical activity and stress, namely striated muscles, MG53 is a tripartite motif (TRIM) protein that presides over the mechanisms of plasma membrane repair. It facilitates the nucleation of intracellular transport vesicles to sites of membrane injury to form a mending patch. A lack of MG53 causes inadequate cell membrane repair that can lead to progressive skeletal myopathy and a decreased regenerative capacity of cardiomyocytes[7,8]. Research efforts have been devoted to translate this rudimentary discovery to clinical applications towards treatment of muscular dystrophy[9–11], acute lung injury[12], and other human diseases, including myocardial infarction [8,10,13,14] and neurodegeneration.

Recognizing the prevalence of metabolic syndrome-mediated tissue regeneration deficiency and MG53’s indispensable function in restoration of membrane integrity, we wondered whether metabolic disorders affect MG53 activity within striated muscles. If metabolic syndrome adversely impacts MG53 function, how can this interaction be elucidated on the molecular scale?

To analyze metabolic disorder-mediated regulation of MG53 activity, we established mice models with metabolic diseases by subjecting wild-type mice to a high-fat diet (HFD) for six months and evaluated the extent of their MG53 expression. Our findings indicate that HFD-induced metabolic defects do not alter MG53 levels within skeletal and cardiac muscles. However, the mice inflicted with metabolic syndrome do exhibit a diminution of MG53 in blood circulation. More interestingly, the HFD treatment caused a localization of MG53 around mitochondria, a unique intracellular distribution.

## Materials and Methods

### Generation of Mice Models with Metabolic Syndrome

C57/BL6 wild type mice (4 weeks age) were purchased from Jackson laboratory in Maine. They were bred within vented cages with 12 hours of light/dark cycle within a temperature controlled environment and had free access to food and water. Feeding for the first 4 weeks consisted of a normal diet (ND) (13.5% fat, 28.5% protein, 58% carbohydrates); afterwards, eight of the mice were subjected to a high fat diet (HFD) (60.9% fat, 18.3% protein, 20.1% carbohydrates) for 6 months prior to any lab testing. The experimental protocol was approved by the Institutional Animal Care and Use Committee at The Ohio State University Wexner Medical Center.

### Glucose and Insulin Tolerance Test

For the glucose tolerance test, a 10% solution of D-glucose in phosphate buffer saline (PBS) was prepared the night before and the mice were fasted for a period of 16 hours. Injections of glucose were made to the intraperitoneal region (1 g/kg according to body mass) and the resultant blood glucose levels were measured at 0, 30, 60 and 120 minutes after glucose distribution. Blood samples were collected from a tail vein and the mice’s glucose concentrations were calculated via an automatic glucose monitor.

For the insulin tolerance test, a 100 U/mL insulin solution (Sigma-Aldrich Co.) was prepared the morning of the procedure and the mice were fasted for a period of 6 hours. Injections of insulin were also made to the intraperitoneal region (0.75 U/kg according to body mass) and the resultant blood glucose levels were measured at 0, 30, 60, and 90 minutes after insulin administration. Blood samples were collected from a tail vein and the mice’s glucose concentrations were calculated via an automatic glucose monitor.

### Lipid Profiling

Forty μL plasma samples derived from the mice of each experimental group were analyzed for lipid concentration using an Alere Cholestech LDX commercial kit (Alere, San Diego, CA). Total cholesterol and low density lipoprotein (LDL) concentrations were measured.

### Western Blotting

Gastrocnemius muscle tissues and hearts of the mice were isolated and lysed with an RIPA buffer (25 mM Tris-HCl of pH 7.6, 150 mM NaCl, 1% NP-40, 1% sodium deoxycholate, 0.1% SDS) supplemented by a protease inhibitor cocktail (Roche, Applied Science). Plasma samples were collected in heparinized tubes and centrifuged at 1000 g for 20 minutes, as described in our previous publication[9]. Twenty-five μg of total protein (muscle samples) and one μL of plasma were loaded and separated on 8% SDS polyacrylamide gels, respectively. Proteins were then transferred to a polyvinylidene difluoride membrane and probed with a primary antibody against MG53 and horseradish peroxidase conjugated secondary antibody. Peroxidase activity was developed with ECL kits (Pierce). Panceau S staining was used to indicate equality in sample loading.

### Immunofluorescence Staining

Paraffin embedded tibialis anterior (TA) muscle tissues of 4 μm thicknesses were used for the immunofluorescence staining of MG53. A monoclonal rabbit-against-mouse MG53 primary antibody and an Alexa-647-conjugated goat-against-rabbit secondary antibody were used to determine the localization of MG53 distribution. Transverse and longitudinal sections of the TA muscle were examined through confocal microscopy using a Zeiss 780 system.

### Statistical Analysis

Statistical significance of differences between the groups was determined employing two sample t-tests. All data is expressed as mean ± standard deviation. P-values of less than 0.05 were considered significant.

## Results

### HFD induces metabolic disorders in mice

**Fig 1:**
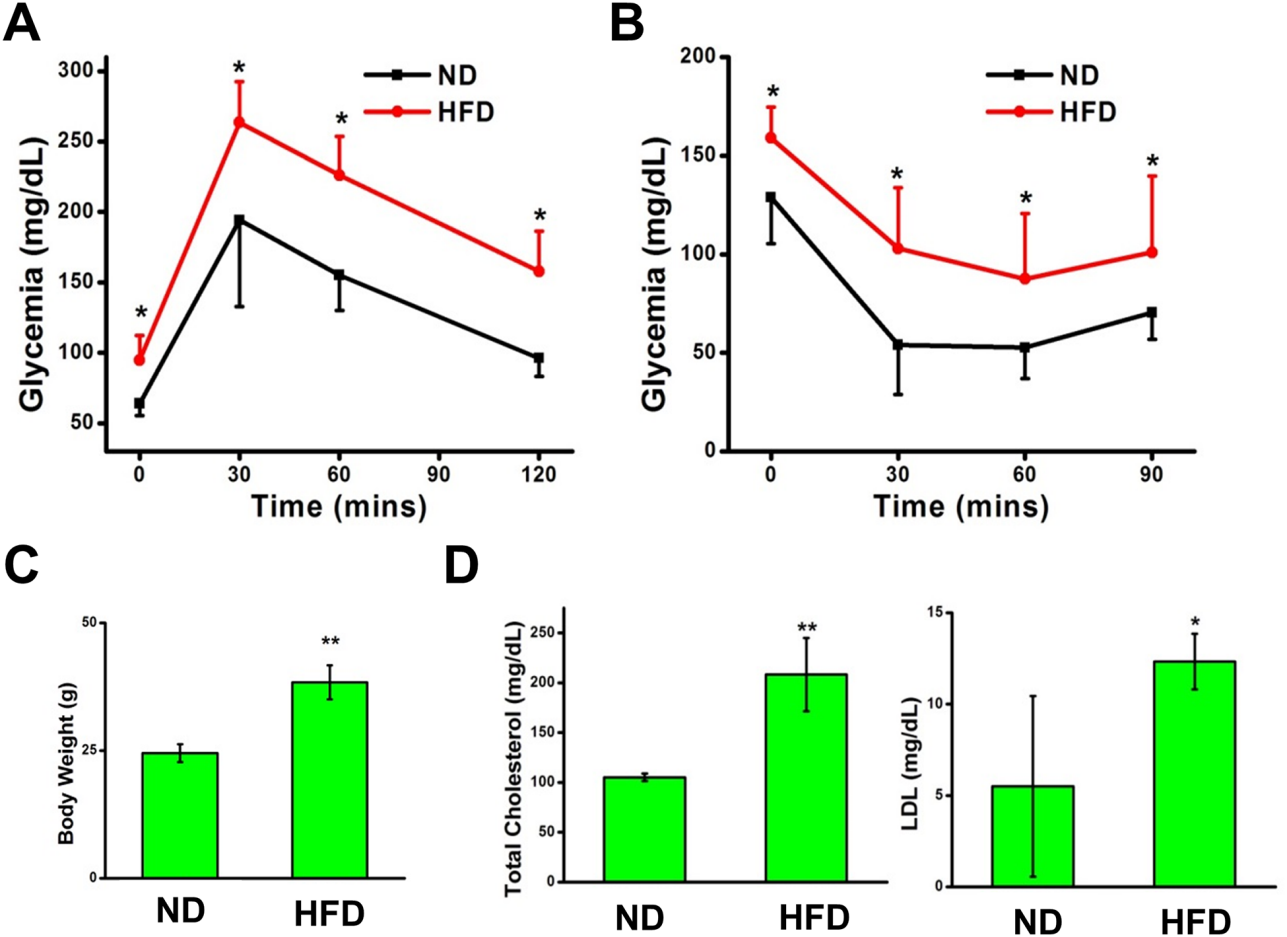
HFD-treated mice exhibit metabolic syndrome. (**A**) HFD-treated mice are glucose intolerant and (**B**) insulin resistant. n = 5 normal diet (ND), n = 8 high fat diet (HFD). (**C**) HFD induces mice obesity. (**D**) Lipid profiling shows elevated total cholesterol and low-density lipoprotein (LDL) levels in HFD-induced mice. The results are presented as mean ± SEM. * p<0.05; ** p<0.01.

To establish the validity of our mice models with metabolic syndrome and the efficacy of the six month HFD treatment, we assessed four vital parameters that are indicative of metabolic disorders: obesity, glucose intolerance, insulin insensitivity, and high blood cholesterol. The mice subjected to HFD treatment exhibit obesity with a significantly larger mean body mass than that of the mice treated with a ND (Fig 1C). The response of each mouse to an injection of glucose (1 g/kg according to body mass) was analyzed through a glucose tolerance test. The mice on the HFD exhibited an inability to cope with elevations of serum glucose concentration, as opposed to the normally fed mice which were able to restore homeostasis to the blood stream in response to hyperglycemia (Fig 1A). Area-under-curve comparisons reveal statistically significant differences between the glycemic regulatory capabilities of the mice treated with a HFD and the mice fed with a ND. Furthermore, the results of the insulin tolerance test show that the HFD mice developed insulin resistance (Fig 1B). The mice subjected to normal conditions were able to successfully utilize injected insulin to precipitate the uptake of circulating serum glucose, effectively lowering glycemia, while the blood glucose levels of the HFD-treated mice remained elevated, a testament to their insulin insensitivity (Fig 1B). Blood lipid concentration was then assessed through serum lipid profiling. The mice treated with a HFD exhibited higher levels of total cholesterol and low-density lipoprotein (LDL) than the mice subjected to a ND (Fig 1D). These data serve to verify the generation of mice models exhibiting metabolic syndrome and to confirm the validity of our succeeding experiments.

### HFD feeding does not alter MG53 expression in striated muscles, but reduces MG53 level in blood circulation

**Fig 2:**
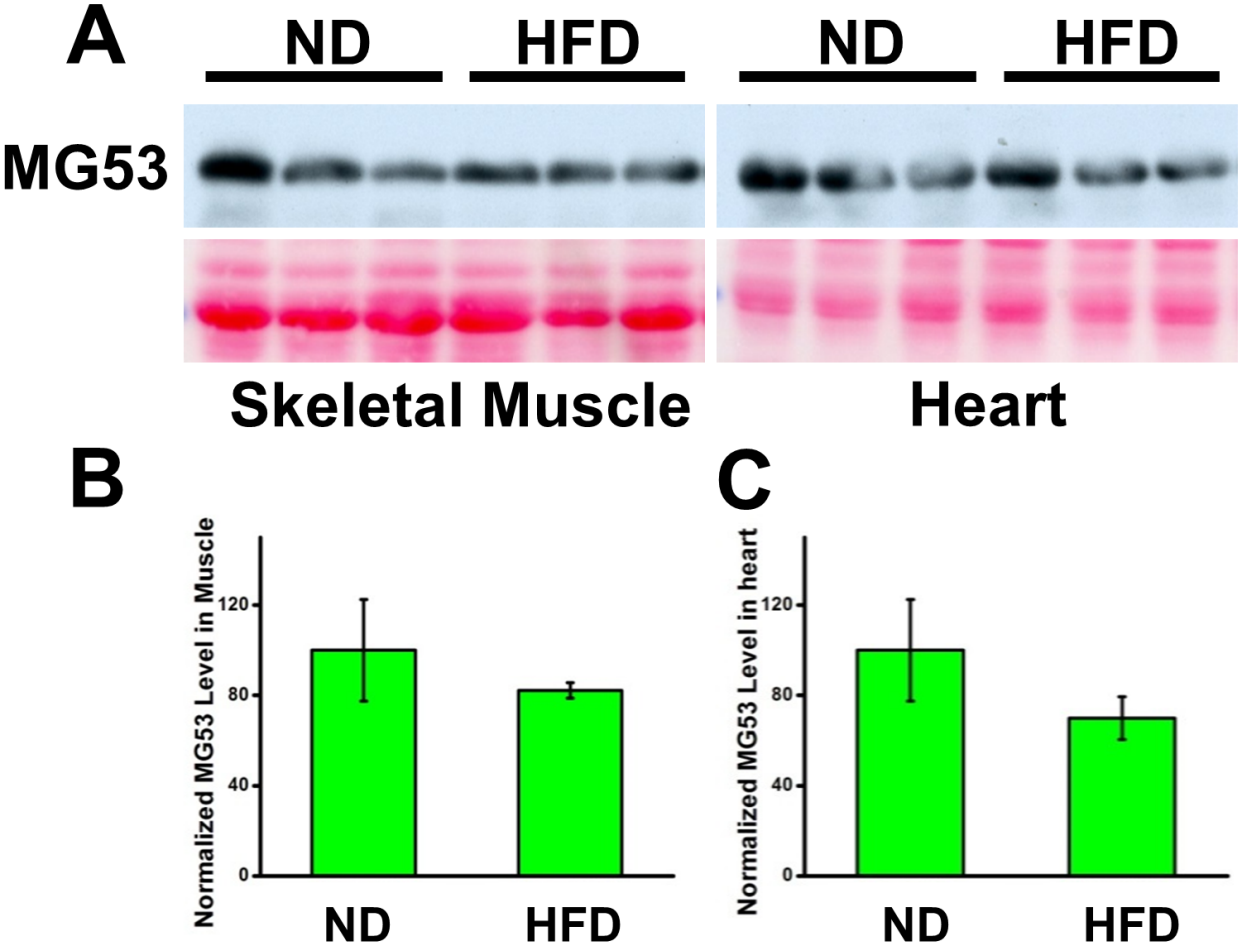
HFD treatment does not alter MG53 expression in striated muscle. (**A**) Western blotting of gastrocnemius and heart muscles. Ponceau red staining shows sample loading equality. Quantifications of western blot densities exhibit no significant difference in MG53 expression between ND (n = 5) and HFD (n = 7)-treated mice in skeletal (**B**) and heart muscle (**C**). The results are presented as mean ± SEM.

Once valid mice models with metabolic diseases were established, we proceeded to test our initial question: whether MG53 activity is altered by metabolic disorders. Our assessment involves the comparison of MG53 presence in skeletal and cardiac muscle tissue between the mice with metabolic syndrome and their normal counterparts. For this analysis, we performed western blotting on the gastrocnemius and heart muscle tissues of each mouse with a monoclonal antibody against MG53 (Fig 2A). The densities of the generated blots were quantified, revealing an insignificant difference between the experimental and control group (Fig 2B and 2C). This finding indicates that intracellular MG53 expression does not change with the manifestation of metabolic diseases.

**Fig 3:**
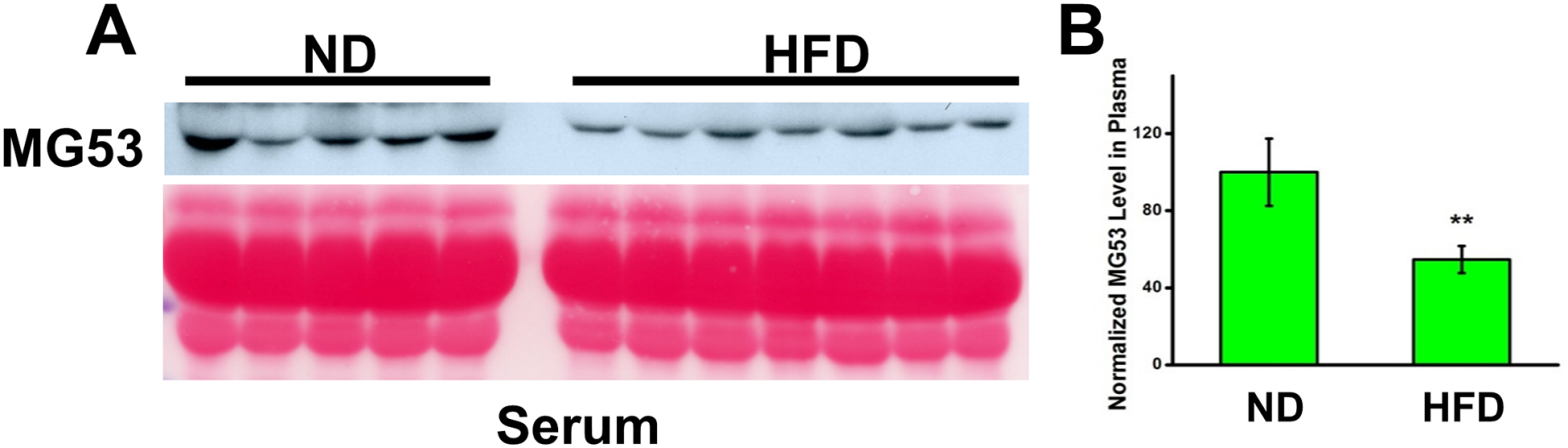
HFD treatment reduces the level of MG53 in blood circulation. (A) Western blotting MG53 levels in serum of ND (n = 5) and HFD (n = 7)-treated mice. (B) Quantification of western blot densities reveals reduced MG53 serum levels in mice on HFD. The results are presented as mean ± SEM. ** p<0.01.

Understanding that this assessment is not representative of MG53’s complete capacity due to the presence of circulating MG53 within the serum, we conducted a western blot analysis for MG53 expression on peripheral blood samples of the mice of each treatment group (Fig 3A). We found that the mice exhibiting metabolic syndrome actually possessed reduced levels of MG53 in their blood circulation than their normal counterparts. Quantification of the western blot densities reveals a significantly lower MG53 serum level in the mice with metabolic disorders (Fig 3B). Together, these data indicate that the presence of metabolic syndrome, induced by HFD feeding, diminishes overall MG53 function, despite its static intracellular concentration, due to its reduction in blood circulation.

### HFD alters subcellular localization of MG53 in skeletal muscles

**Fig 4:**
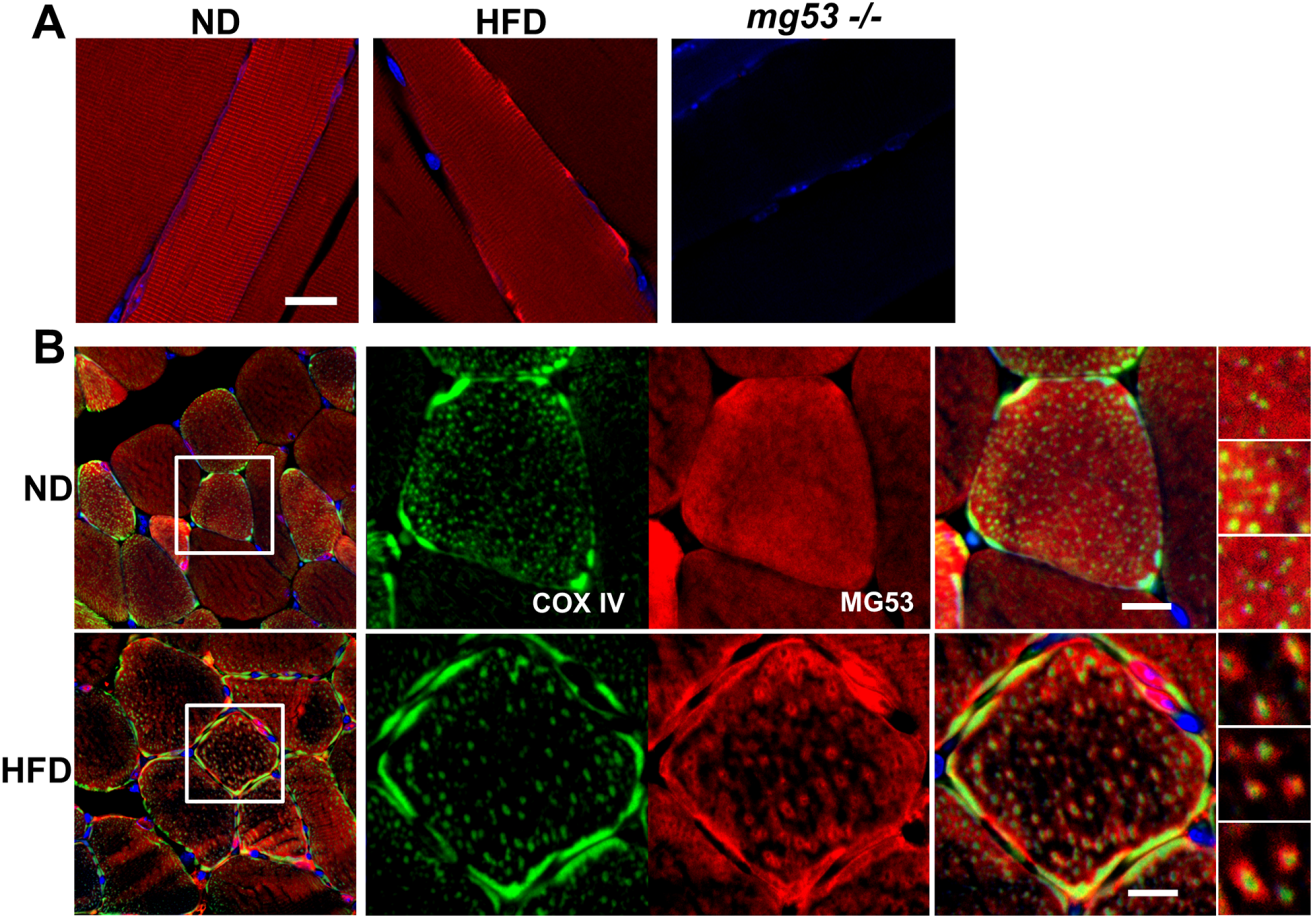
HFD treatment induces MG53 localization to the plasma membrane and mitochondria in skeletal muscle. (A) IHC staining of longitudinal sections of skeletal muscle reveals striated patterns of MG53 in ND-treated mice (left). HFD treatment results in the aggregation of MG53 near the plasma membrane (middle). mg53-/- muscle cells show no staining (right). Scale bar: 20 μm. (B) Cross-sections of TA muscle tissue reveal punctated distribution of MG53 inside the muscle fibers of HFD-treated mice (lower panels), which was not witnessed in those subjected to normal conditions (upper panels). COX IV staining (green) suggests the possibility of MG53 (red) localization around mitochondria, since COX IV positive signals were detected in the center of MG53 punctates (please see the right panels with high magnification). Scale bar: 10 μm.

Another important aspect of MG53 activity involves its intracellular behavior, which can be largely determined by its distribution within the striated muscles. To assay this parameter, we performed an immunhistochemical analysis on TA muscle tissues extracted from the mice of each treatment group. The skeletal muscles were stained with a monoclonal rabbit-against-mouse MG53 primary antibody and an Alexa-647-conjugated goat-against-rabbit secondary antibody. Longitudinal images of these tissues generated through confocal microscopy reveal that metabolic syndrome prompts an aggregation of MG53 near the sarcolemma, evidenced by the very prominent red signals along the edge of the muscle fibers of the mice with metabolic disorders (Fig 4A, middle panel). In contrast, a normal, striated pattern of MG53 distribution can be observed in the TA muscle tissues of the normal mice (Fig 4A, left panel). The validity of the generated pictures was confirmed by the stainless images of tissues derived from MG53 deficient (*MG53* -/-) mice (Fig 4A, right panel).

The precipitation of MG53 amassment to the sarcolemma by the HFD treatment is corroborated by cross-sectional images of the striated muscle tissues, where highlighted red segments appear to perforate the TA muscle fibers of mice with metabolic disorders along the plasma membrane (Fig 4B, bottom middle panel, red channel). However, these pictures reveal a more interesting scene. While the ND mice exhibit a diffused dispersion of intracellular MG53, illustrated by the uniform redness of their muscle fibers (Fig 4B, top middle panel), the HFD-fed mice display a punctated distribution of intra-fibrillar MG53, indicated by the small red clumps scattered throughout the TA muscle fibers of the mice with metabolic syndrome (Fig 4B, bottom middle panel). To elucidate this phenomenon, we co-probed the samples with COX IV, a mitochondria marker (Fig 4B, green channels). Consequently, we discovered that the observed clusters of MG53 are fixed around COX IV positive signals within the muscle fibers of the mice with metabolic disorders. This finding suggests that HFD-induced metabolic syndrome precipitates MG53 localization around mitochondria which are denoted by the intracellular COX IV positive signals.

## Discussion and Conclusion

Our data indicate that intracellular MG53 expression in striated muscles remains unchanged with the manifestation of metabolic syndrome, induced by a HFD treatment, in mice. However, the concentration of MG53 in blood circulation is diminished in mice exhibiting metabolic disorders. Additionally, the HFD treatment induced a unique intracellular distribution of MG53 aggregated around mitochondria. Overall, these findings support that metabolic syndrome adversely impacts MG53 expression and function.

Researchers have previously analyzed MG53’s role in the development of metabolic disorders[15,16]. In particular, Song et al. indicted MG53 as a perpetrator of metabolic syndrome, citing its participation in downregulating insulin receptor substrate-1 (IRS-1) as a problematic source of insulin resistance [16]. As a member of the TRIM family, MG53 possesses a RING finger domain with E3-ligase activity, enabling it to execute the ubiquitination of specific target proteins. Song et al. criticize this property of MG53 that prompts the ubiquitin-dependent degradation of IRS-1. They suggest that “MG53 suffices to trigger muscle insulin resistance and metabolic syndrome sequentially”, observing that MG53 overexpression precedes the onset of metabolic disorders. As well, they claim that “MG53 expression is markedly elevated in models of insulin resistance” and that “its abundance universally increases in high-fat diet (HFD)-induced obese mice, *db/db* diabetic mice, [and] spontaneously hypertensive rats”. Our data directly contradicts this finding. We discovered that MG53 expression within skeletal and cardiac muscles does not change in mice exhibiting metabolic syndrome. Moreover, another group of researchers, Yi et al., assert that “altered MG53 expression does not serve as a causative factor for the development of metabolic disorders”, indicated by “muscle samples derived from human diabetic patients and mice with insulin resistance [that] show normal expression of MG53”[15]. Our findings are also consistent with many others’, including those of Yuan et al.[17], Xu et al.[18], and Ma et al.[19], which show that a high cholesterol diet treatment does not induce MG53 overexpression, despite the obvious presence of metabolic disorders.

Even if an elevation of MG53 expression is incurred by a HFD treatment, as demonstrated by Song et al., the effect of IRS-1 downregulation in the manifestation of metabolic disorders is dubious. This is due to the existence of three other homologous proteins in the IRS family, IRS-2, IRS-3, and IRS-4, each of which possess a distinct physiological role in insulin signal transduction. Studies by Terauchi et al.[20] and Tamemoto et al.[21] show that an absence of IRS-1 is not sufficient to induce type II diabetes, evidenced by the lack of diabetic phenotypes in *Irs1* ^*-/-*^ mice. IRS-3 deficiency also does not alter glycemic regulatory capabilities and *Irs4* single knockout mice only exhibit mild glucose intolerance[22]. IRS-2 under expression may result in the development of diabetes. However, this is through a defect in ß-cell proliferation, and not a result of insufficient glucose uptake by striated muscle cells[23,24]. Findings from previous literature indicate that mice with ablation of IRS-1 and IRS-2 in skeletal muscle cells and *Irs1* ^*-/-*^ */Irs4* ^*-/-*^ double knockout mice do not exhibit abnormalities in glucose homeostasis[22,25]. Interestingly, only through a combination of IRS-1 and IRS-3 deficiency does a manifestation of diabetic phenotypes result, demonstrated by Laustsen et al., indicating that IRS-1 and IRS-3 serve overlapping physiological functions in insulin signal transduction[22]. Thus, MG53-mediated IRS-1 downregulation cannot possibly induce type II diabetes through insulin resistance as the existence of IRS-3 compensates for IRS-1 absence.

Although our results display that a HFD treatment does not alter MG53 expression in skeletal and cardiac muscle tissues, HFD-induced metabolic syndrome does procure a decrease in extracellular, circulating MG53. This phenomenon may contribute to the tissue repair defects observed in diabetic patients[5,6]. A study by Dr. Paul McNeil’s group demonstrated that plasma membrane repair defects might result from diabetic complications[26]. Individuals inflicted with metabolic disorders are unable to effectively utilize MG53’s membrane restoration potential due to its inadequate expression in serum circulation. Thus, the compromised tissue regeneration capacity of diabetic patients may be rectified by elevating MG53 in the serum.

More interestingly, we found that chronic exposure to metabolic syndrome precipitated subcellular MG53 localization around mitochondria. This finding indicates that MG53 may play a guardian role in mitochondrial membrane protection. The HFD feeding induced an elevation of mitochondria activity, resulting in the excessive production of reactive oxygen species (ROS), adverse byproducts of cell respiration. The oxidative stress caused by the accumulation of such chemically reactive molecules may have caused significant damage to the mitochondrial membrane. Upon injury, the mitochondria may have exposed a signal that prompted MG53 movement for membrane repair. Protection of mitochondrial welfare could necessitate the observed MG53 aggregation. To our knowledge, this is a novel finding as MG53’s interaction with subcellular structures has never before been explored, particularly regarding the mitochondria.

In the future, we seek to dissect the function of MG53 in protecting against mitochondria injury by identifying the specific molecular mechanisms underlying MG53 translocation to the mitochondrial membrane. We will also determine the specific molecular mechanisms that underlie metabolic syndrome-mediated reduction of circulating MG53. There are several possible explanations for the diminished MG53 levels in blood circulation. First, MG53 uptake by damaged tissues could have been enhanced in the mice exhibiting HFD-induced metabolic syndrome. It has been established that the presence of metabolic disorders is associated with multiple tissue injury. Since this is the case, MG53 could have been siphoned from the serum to protect injured muscle tissues in the HFD-fed mice. Second, the HFD treatment may have inhibited MG53 secretion from skeletal and cardiac muscle tissues. We will study the impaired exocytotic pathways of MG53 in striated muscle cells if this is the case. Alternatively, the reduction of circulating MG53 may have been procured by an increase in MG53 degradation in the mice inflicted with metabolic syndrome. If this were true, the enzyme involved in such denaturation will be analyzed. Another potential source of the decreased MG53 serum levels in the mice treated with a HFD involves an elevation of MG53 clearance by the kidney. It was recently found that the half-life of serum MG53 is identical in mice, rats, and dogs, suggesting that the pharmacokinetic properties of MG53 are largely determined by glomerular filtration. The reduced levels of circulating MG53 may reflect kidney dysfunction in the mice with metabolic disorders. Since it is widely recognized that the manifestation of metabolic syndrome adversely impacts kidney function, we suspect that the HFD treatment may have increased kidney-mediated clearance of MG53 by reducing reabsorption.

Overall, our findings indicate that the manifestation of metabolic syndrome alters MG53 activity by reducing its extracellular expression in the serum and causing it to aggregate around mitochondria within striated muscle cells. The tissue regeneration defect observed in individuals inflicted with metabolic disorders can be explained by the metabolic syndrome-mediated downregulation of MG53 in serum. Thus, therapeutic approaches to elevate circulating MG53 may be a novel means to treat the compromised tissue repair capacity of diabetic patients.

## References

[1] Cornier MA, Dabelea D, Hernandez TL, Lindstrom RC, Steig AJ, Stob NR, et al The metabolic syndrome. Endocrine reviews. 2008;29(7):777–822. 10.1210/er.2008-0024 18971485 PMC5393149

[2] Isomaa B, Almgren P, Tuomi T, Forsen B, Lahti K, Nissen M, et al Cardiovascular morbidity and mortality associated with the metabolic syndrome. Diabetes care. 2001;24(4):683–9. 11315831 10.2337/diacare.24.4.683

[3] Weiss R, Dziura J, Burgert TS, Tamborlane WV, Taksali SE, Yeckel CW, et al Obesity and the metabolic syndrome in children and adolescents. The New England journal of medicine. 2004;350(23):2362–74. 15175438 10.1056/NEJMoa031049

[4] Moller DE, Kaufman KD. Metabolic syndrome: a clinical and molecular perspective. Annual review of medicine. 2005;56:45–62. 15660501 10.1146/annurev.med.56.082103.104751

[5] Giacco F, Brownlee M. Oxidative stress and diabetic complications. Circulation research. 2010;107(9):1058–70. 10.1161/CIRCRESAHA.110.223545 21030723 PMC2996922

[6] Forbes JM, Cooper ME. Mechanisms of diabetic complications. Physiological reviews. 2013;93(1):137–88. 10.1152/physrev.00045.2011 23303908

[7] Cai C, Masumiya H, Weisleder N, Matsuda N, Nishi M, Hwang M, et al MG53 nucleates assembly of cell membrane repair machinery. Nat Cell Biol. 2009;11(1):56–64. 10.1038/ncb1812 19043407 PMC2990407

[8] Wang X, Xie W, Zhang Y, Lin P, Han L, Han P, et al Cardioprotection of ischemia/reperfusion injury by cholesterol-dependent MG53-mediated membrane repair. Circ Res. 2010;107(1):76–83. 10.1161/CIRCRESAHA.109.215822 20466981

[9] Weisleder N, Takizawa N, Lin P, Wang X, Cao C, Zhang Y, et al Recombinant MG53 protein modulates therapeutic cell membrane repair in treatment of muscular dystrophy. Sci Transl Med. 2012;4(139):139ra85 10.1126/scitranslmed.3003921 22723464 PMC3777623

[10] He B, Tang RH, Weisleder N, Xiao B, Yuan Z, Cai C, et al Enhancing muscle membrane repair by gene delivery of MG53 ameliorates muscular dystrophy and heart failure in delta-Sarcoglycan-deficient hamsters. Mol Ther. 2012;20(4):727–35. 10.1038/mt.2012.5 22314291 PMC3321592

[11] Zhu H, Lin P, De G, Choi KH, Takeshima H, Weisleder N, et al Polymerase transcriptase release factor (PTRF) anchors MG53 protein to cell injury site for initiation of membrane repair. J Biol Chem. 2011;286(15):12820–4. 10.1074/jbc.C111.221440 21343302 PMC3075629

[12] Jia Y, Chen K, Lin P, Lieber G, Nishi M, Yan R, et al Treatment of acute lung injury by targeting MG53-mediated cell membrane repair. Nature communications. 2014;5:4387 Epub 2014/07/19. 10.1038/ncomms5387 25034454 PMC4109002

[13] Zhang Y, Lv F, Jin L, Peng W, Song R, Ma J, et al MG53 participates in ischaemic postconditioning through the RISK signalling pathway. Cardiovascular research. 2011;91(1):108–15. 10.1093/cvr/cvr029 21285295 PMC3112015

[14] Cao CM, Zhang Y, Weisleder N, Ferrante C, Wang X, Lv F, et al MG53 constitutes a primary determinant of cardiac ischemic preconditioning. Circulation. 2010;121(23):2565–74. 10.1161/CIRCULATIONAHA.110.954628 20516375

[15] Yi JS, Park JS, Ham YM, Nguyen N, Lee NR, Hong J, et al MG53-induced IRS-1 ubiquitination negatively regulates skeletal myogenesis and insulin signalling. Nature communications. 2013;4:2354 10.1038/ncomms3354 23965929 PMC3941707

[16] Song R, Peng W, Zhang Y, Lv F, Wu HK, Guo J, et al Central role of E3 ubiquitin ligase MG53 in insulin resistance and metabolic disorders. Nature. 2013;494(7437):375–9. 10.1038/nature11834 23354051

[17] Yuan H, Niu Y, Liu X, Yang F, Niu W, Fu L. Proteomic analysis of skeletal muscle in insulin-resistant mice: response to 6-week aerobic exercise. PloS one. 2013;8(1):e53887 10.1371/journal.pone.0053887 23326526 PMC3541238

[18] Xu Y, Ma LL, Zhou C, Zhang FJ, Kong FJ, Wang WN, et al Hypercholesterolemic myocardium is vulnerable to ischemia-reperfusion injury and refractory to sevoflurane-induced protection. PloS one. 2013;8(10):e76652 10.1371/journal.pone.0076652 24124583 PMC3790738

[19] Ma LL, Zhang FJ, Qian LB, Kong FJ, Sun JF, Zhou C, et al Hypercholesterolemia blocked sevoflurane-induced cardioprotection against ischemia-reperfusion injury by alteration of the MG53/RISK/GSK3beta signaling. International journal of cardiology. 2013;168(4):3671–8. 10.1016/j.ijcard.2013.06.037 23856444

[20] Terauchi Y, Iwamoto K, Tamemoto H, Komeda K, Ishii C, Kanazawa Y, et al Development of non-insulin-dependent diabetes mellitus in the double knockout mice with disruption of insulin receptor substrate-1 and beta cell glucokinase genes. Genetic reconstitution of diabetes as a polygenic disease. The Journal of clinical investigation. 1997;99(5):861–6. 9062343 10.1172/JCI119250 PMC507893

[21] Tamemoto H, Kadowaki T, Tobe K, Yagi T, Sakura H, Hayakawa T, et al Insulin resistance and growth retardation in mice lacking insulin receptor substrate-1. Nature. 1994;372(6502):182–6. 7969452 10.1038/372182a0

[22] Laustsen PG, Michael MD, Crute BE, Cohen SE, Ueki K, Kulkarni RN, et al Lipoatrophic diabetes in Irs1(-/-)/Irs3(-/-) double knockout mice. Genes & development. 2002;16(24):3213–22. 12502742 10.1101/gad.1034802 PMC187498

[23] Withers DJ, Gutierrez JS, Towery H, Burks DJ, Ren JM, Previs S, et al Disruption of IRS-2 causes type 2 diabetes in mice. Nature. 1998;391(6670):900–4. 9495343 10.1038/36116

[24] Kubota N, Tobe K, Terauchi Y, Eto K, Yamauchi T, Suzuki R, et al Disruption of insulin receptor substrate 2 causes type 2 diabetes because of liver insulin resistance and lack of compensatory beta-cell hyperplasia. Diabetes. 2000;49(11):1880–9. 11078455 10.2337/diabetes.49.11.1880

[25] Long YC, Cheng Z, Copps KD, White MF. Insulin receptor substrates Irs1 and Irs2 coordinate skeletal muscle growth and metabolism via the Akt and AMPK pathways. Molecular and cellular biology. 2011;31(3):430–41. 10.1128/MCB.00983-10 21135130 PMC3028618

[26] Howard AC, McNeil AK, Xiong F, Xiong WC, McNeil PL. A novel cellular defect in diabetes: membrane repair failure. Diabetes. 2011;60(11):3034–43. 10.2337/db11-0851 21940783 PMC3198060

